# Dynamic analysis of liquid annular seals with herringbone grooves on the rotor based on the perturbation method

**DOI:** 10.1098/rsos.180101

**Published:** 2018-06-06

**Authors:** Lulu Zhai, Zhang Zhenjie, Chi Zhonghuang, Guo Jia

**Affiliations:** 1 The Province Key Laboratory of Fluid Transmission Technology, Zhejiang Sci-Tech University, Hangzhou 310018, People's Republic of China; 2 Zhejiang Institute of Mechanical & Electrical Engineering Co., Ltd, Hangzhou 310000, People's Republic of China

**Keywords:** liquid annular seal, herringbone grooves, perturbation method, dynamic characteristics

## Abstract

Annular seals have significant effects on the hydraulic and rotordynamic performances of turbomachinery. In this paper, an analysis method for calculating the leakage flow rates and dynamic characteristics of liquid annular seals with herringbone grooves on the rotor is proposed and verified. Leakage flow rates and dynamic characteristics of the model seals under different operating conditions are theoretically analysed and compared with those of plain and spiral-grooved seals of the same size. In addition, the influence of geometric parameters such as spiral angle and the lengths of the constituent parts on the sealing and rotordynamic coefficients of seals with herringbone grooves are also discussed. The results show that seals with herringbone grooves have better sealing performance, while providing better support actions and damping characteristics than the other two seal types under the same operating conditions. The seal geometric parameters including spiral angle, the lengths of the constituent parts and the clearance value have a significant influence on the dynamic characteristics of seals with herringbone grooves.

## 1. Introduction

Annular liquid seals are primarily used to control leakage in turbomachinery, especially in multi-stage centrifugal pumps such as boiler feed pumps and other heavy-duty pumps. The forces generated in these seal paths have significant effects on the rotordynamic characteristics of multi-stage centrifugal pumps. A rotor–bearing–seal system can improve the stability and rigidity of the whole shafting system [1]. An effective method of improving the stabilizing effect of annular seals is engraving some grooves either on the rotor or on the inner surface of the stator, such as straight labyrinth seals, stepped labyrinth seals, spiral-grooved seals and herringbone-grooved seals. Similar to spiral-grooved seals, the inward pumping action of herringbone grooves will minimize the leakage rate as well as generate load capacity and stiffness (table 1).

**Table 1.** Nomenclature.

	Roman letters		
*C*	mean radial clearance of the middle plain part	*Q*	leakage flow rate
*C_h_*	direct damping	*R*	seal radius
*c_h_*	cross-coupled damping	*R*_0_	amplitude of seal harmonic motion
*C_l_*_0_	mean radial clearance of the spiral part	*r*_0_	non-dimensional amplitude of seal harmonic motion
*I*_s_	thread number	*T*	groove depth
*K_h_*	direct stiffness	*ū_z_*	non-dimensional axial velocity
*k_h_*	cross-coupled stiffness	*ū_θ_*	non-dimensional circumferential velocity
*L*	length	*z*	axial coordinate
*L_l_*	land width of the spiral-grooved part in the *η-*direction		
*L_g_*	groove width of the spiral-grooved part in the *η-*direction		
*M_h_*	direct added mass		
*P, ΔP*	pressure difference		
*P*_b1_	boundary pressure at the inlet of the middle plain part		
*P*_b2_	boundary pressure at the outlet of the middle plain part		
*P*_in_	inlet pressure of the seal with herringbone grooves		
*P*_out_	outlet pressure of the seal with herringbone grooves		
Greek letters			
*α*	spiral angle	*ε*	perturbation coefficient
λ	friction coefficient	*γ*	divergent flow angle
*μ*	dynamic viscosity	*υ*	kinematic viscosity
*ξ*	pressure loss coefficient	*ρ*	fluid density
*Ω*	whirling speed	*ω*	rotational speed
subscript			
0	zeroth-order solutions	*s*	spiral part
1	first-order solutions	ups	upstream spiral part
downs	downstream spiral part	*x*	*x* coordinate
*g*	groove portion	*y*	*y* coordinate
*h*	annular seal with herringbone grooves	*z*	axial direction
in	inlet pressure loss	*ζ*	*ζ-*direction
*l*	land portion	*η*	*η-*direction
*p*	middle plain part	*θ*	circumferential direction
*r*	radial direction	λ	friction pressure loss
out	outlet pressure loss		

For decades, researchers and engineers have carried out many studies on the sealing and dynamic property predictions of annular plain seals and seals with different kinds of grooves to facilitate superior rotordynamic characteristics while providing good leakage control. Childs [2] performed a finite-length analysis for annular plain seals based on Hirs' lubrication equations and the bulk-flow model proposed by Black. This analysis method has been the most widely used in engineering. Nelson & Nguyen [3,4] developed a new solution method that was different from Childs's model that used fast Fourier transforms (FFTs) to determine the effects of eccentricity on the rotordynamic coefficients of plain annular seals. Kostyuk [5] performed the first comprehensive analysis of the aerodynamic forces of gas labyrinth seals excluding the influence of area change due to eccentricity on cross-coupled forces. Then, Iwatsubo *et al*. [6,7] refined Kostyuk's model by introducing the time dependency of area change. Childs & Scharrer [8] presented a unified and comprehensive derivation of the motion equations for compressible flow within straight labyrinth seals taking account of the area change in the circumferential direction due to eccentricity. The equations were solved by the perturbation method, and the predicted rotordynamic coefficients were within 25% of the experimental results. Nordmann *et al*. [9] and Kim & Childs [10] studied the leakage and dynamic characteristics of seals with circumferential and spiral grooves by introducing equivalent roughness coefficients in both the circumferential and axial directions based on Hirs' turbulent lubrication theory. The theoretical predictions mentioned above were performed based on the single control-volume method and perturbation analysis. Subsequently, Iwatsubo *et al*. [11–13] theoretically analysed the static and dynamic characteristics of liquid annular seals with parallel grooves, spiral grooves and double spiral grooves on a rotor using a detailed two-control-volume description of the seal in the land and groove sections. Wyssmann *et al*. [14] and Scharrer & Childs [15,16] supplemented the two-control-volume approach and applied it to gas labyrinth seals. The two-control-volume method is widely used in engineering. Florjancic [17] and Marquette & Childs [18] developed a three-control-volume approach for liquid circumferentially grooved seals, featuring an excellent description of the flow inside the groove cavity. The predictions are excellent for leakage as well as rotordynamic coefficients. Muszynska [19] and Muszynska & Bently [20] proposed a nonlinear seal fluid dynamic force model that considered the circulating velocity as the key factor affecting the stability of the rotor system. Fleming [21] applied the principle of herringbone-grooved journal bearings to the case of a liquid seal disc with herringbone grooves running under a finger seal pad, and the calculation results showed that significant stiffness and load capacity can be supplied by herringbone grooves. Zhou *et al*. [22–24] proposed the dynamic analysis method for a double-disc rotor–seal system, rotor–seal–bearing system and the pump–turbine–shafting system based on Muszynska's nonlinear seal dynamic analysis. Zhai *et al*. [25] developed a theoretical analysis method for the leakage rate and dynamic characteristics of herringbone-grooved liquid seals based on the method of Iwatsubo and Childs. The analysis method is validated through comparing the predicted leakage rates and hydraulic forces within five test seals with the experimental results. Cangioli *et al*. [26] proposed a thermodynamic analysis method by considering the energy equation in the steady-state calculation of the bulk-flow model. The numerical results are validated by a teeth-on-stator labyrinth seal conducted on the test rig of GE Oil and Gas.

Recently, with the development of computational fluid dynamics (CFD), the sealing and dynamic characteristics of annular seals and a dry-gas seal with complex geometries have been investigated using CFD-based methods. Bhattacharya [27], Nielsen [28], Gao & Kirk [29–31], Untaroiu *et al*. [32,33] and Wu *et al*. [34,35] carried out further implementation and validation for the predictions of dynamic forces and leakage rates of seals with different grooves with different length–diameter ratios. Chochua & Soulas [36], Yan *et al*. [37] and Nielsen *et al*. [38] investigated the hole-pattern seal and convergent honeycomb seal using the CFD-based instationary perturbation model and compared the results with the results of experiments and the ISOTSEAL bulk-flow code. Wang *et al*. [39] numerically analysed the turbulence effects [40] and micro-scale effects on the sealing performances of a spiral-grooved dry-gas seal, respectively, using the direct numerical simulation (DNS) method, the Reynolds-averaged Navier–Stokes simulation (RANS) method and a numerical solution of a corrected Reynolds equation.

The improved prediction capabilities come at much higher computational costs. Therefore, CFD-based methods are mainly used only in the research field, while theoretical prediction procedures based on the bulk-flow theory are still the main methods for calculating leakage rates and dynamic characteristics in engineering.

An extensive theoretical prediction method and procedure for the sealing and dynamic performances of liquid annular seals are still needed. Consequently, in this paper an analytical procedure to determine the dynamic characteristics of liquid annular seals with herringbone grooves on a rotor is proposed based on the perturbation method. Moreover, the effects of operating conditions, clearance and groove patterns on the dynamic characteristics of the seals with herringbone grooves are investigated using this method.

## 2. Theoretical analysis

### 2.1. Modelling

**Figure 1.**
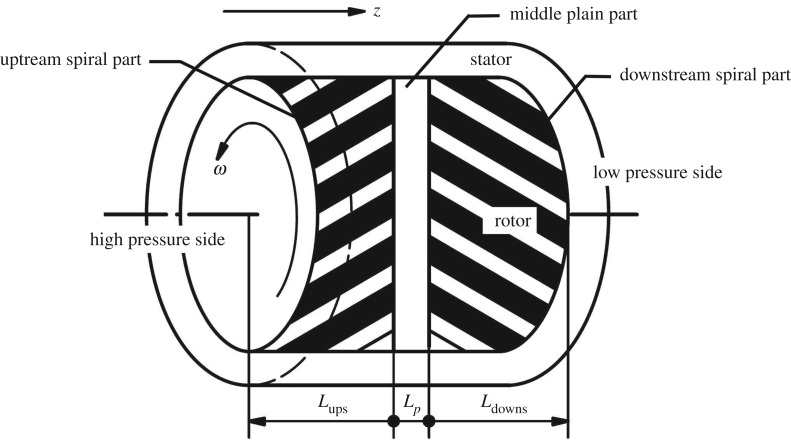
Schematic and geometric parameters of a seal with herringbone grooves on the rotor.

Figure 1 shows the typical configuration of a seal with herringbone grooves on the rotor. As is shown, the herringbone grooves consist of two sets of spiral grooves in two opposite directions. One set is located on the high-pressure side, called the upstream spiral part, and the other is located on the low-pressure side, called the downstream spiral part. There is a narrow middle plain part between the two spiral grooves that functions as a storage area. Generally, the upstream grooves are designed to restrict leakage flow in the operating direction of rotation. The spiral angles of the model seals employed in this paper are less than 15° and the fluid velocity within the seals is assumed to be uniformly distributed along the circumferential direction at the entrance to each part.

Although the geometric shape of the seal is similar to that of a journal bearing, there are many differences between bearings and seals. The major difference is the value of the clearance to radius ratio (i.e. the C/R ratio). For seals, the ratio is generally of the order of 0.03 or more, while it is 0.001 for bearings. These enlarged clearances, combined with large pressure differences and low-viscosity liquids, make the flow in the clearance paths highly turbulent. Hence, the Reynolds equations for annular seals are completely different from those of bearings [41].

### 2.2. Steady-flow velocities and leakage rate of annular seals with herringbone grooves

Fluid flow within a seal with herringbone grooves is composed of three parts: flow within two spiral parts and flow within the middle plain part. The leakage flow rates of each part depend on the pressure gradient for a particular herringbone-grooved seal under certain operating conditions.

#### 2.2.1. Steady-flow characteristics of spiral parts

**Figure 2.**
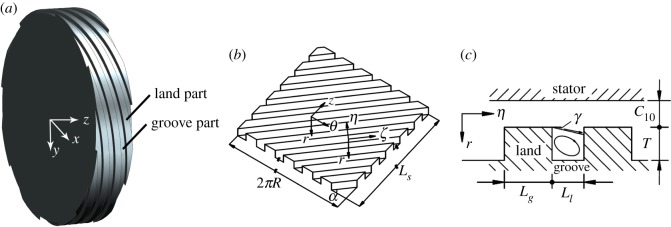
(*a*). Three-dimensional model of the spiral part. (*b*) Expanded figure of a spiral-grooved rotor. (*c*) Cross-sectional view of the spiral part.

The effective pressure differences across the spiral parts are the result of the operating pressure and the pressure induced by the pumping action. For the upstream grooved part, the effective pressure difference is less than the initial one. However, the situation is just the reverse for the downstream grooved part. A *η–r–ζ* coordinate system is built, as shown in figure 2. The spiral part consists of two parts: the land portion and the groove portion. The *η*-direction and the *ζ*-direction are, respectively, set perpendicular and parallel to the groove direction. Flow between the land part and the stator along the *ζ*-direction is approximated as a flow between two parallel plates, and the flow in the groove is approximated as the flow in a rectangular cross-sectional tube. The vortex in the *η*-direction is supposed to diverge with angle *γ* and then go to the next land, as shown in figure 2*c*. Referring to the method proposed by Zhai *et al.* [25], a pressure equilibrium within the groove portion and the land portion in the *η*-direction and the *ζ*-direction between the effective pressure difference and pressure losses induced by wall friction and inlet and outlet pressure losses is applied to determine the leakage flow rates as well as the steady-flow velocities *u_ηl_*_0_, *u_ζl_*_0_, *u_θl_*_0_, *u_zl_*_0_, *u_ηg_*_0_, *u_ζg_*_0_, *u_θg_*_0_ and *u_zg_*_0_.

#### 2.2.2. Steady-flow characteristics of middle plain parts

In principle, axial and circumferential steady-flow velocities of the plain part can be worked out in the same way as those of the grooved part. Note that, although the pressure drop across the plain part consists of three parts (i.e. the inlet, outlet and friction pressure losses), the friction loss term derived from Hirs' equation is one order of magnitude greater than the inlet and outlet losses for a typical seal used in centrifugal pumps. In that case, the friction loss would completely dominate the leakage flow rate [41]. The leakage rate of the plain part excluding the effect of the inlet and outlet pressure losses can be simplified as equation (2.1), where λ_*p*_ is calculated based on Hirs' theory,

2.1 $$Q_{p}=2\pi R\sqrt{\frac{\Delta P_{p}\cdot {C_{p}}^{3}}{\lambda_{p}L_{p}\rho }}.$$

Although the fluid flow within the seal with herringbone grooves is analysed as three individual parts, the velocity distribution of each part has a great influence on the inlet flow conditions of the next part. The leakage flow rate of the three parts should be the same because of mass conservation. Hence, the inlet and outlet pressures of the middle plain part are assumed to be the boundary pressures *P*_b1_ and *P*_b2_. These two boundary pressures can be obtained by solving the leakage equation of the three parts using the traversing method,

2.2 $$Q_{\mathrm{ups}}(P_{\mathrm{in}},P_{b1})=Q_{p}(P_{b2},P_{b1})=Q_{\mathrm{downs}}(P_{b2},P_{\mathrm{out}}),$$

where *P*_in_ and *P*_out_ are, respectively, the inlet and outlet pressures of the whole seal. Thus, static characteristics, including the leakage flow rate and the steady-flow velocity distribution within each of the three parts, will be determined with the given boundary pressures *P*_b1_ and *P*_b2_.

### 2.3. Dynamic forces and characteristics of annular seals with herringbone grooves

Perturbation in the eccentricity ratio *ε* is introduced to linearize the governing equations for each part. The zeroth-order perturbation governing equations describe the steady-flow condition, and the first-order perturbation equations describe the flow conditions due to seal motion. Dynamic forces and coefficients including direct and cross-coupled stiffness, direct and cross-coupled damping and direct added mass coefficients are derived from the pressure-field distribution, which is determined by the first-order perturbation governing equations with boundary pressures given in §2.2.

#### 2.3.1. Dynamic characteristics and forces of the spiral parts

Under the assumptions of ‘fine groove' theory proposed by Iwatsubo [6], non-dimensional first-order perturbation governing equations within the land portion of the spiral-grooved part including the circumferential-momentum equation, axial-momentum equation and continuity equation are as follows:

2.3 $$\left\{ \begin{array}{l} \frac{\partial {\bar{u}}_{zl1}}{\partial \bar{z}}\cdot \frac{\cos\alpha }{L_{l}}-i\frac{1}{R}{\bar{u}}_{\theta l1}=i\frac{R_{0}}{C_{l0}\varepsilon }\frac{1}{R\omega }\left(\frac{\Omega }{C_{l0}}-\frac{{\bar{u}}_{\theta l0}}{C_{l0}R}\right)\\ \frac{\partial {\bar{u}}_{\theta l1}}{\partial \bar{z}}\cdot \frac{\cos\alpha }{L_{l}}+{\bar{u}}_{zl1}\cdot \left[\frac{1}{u_{zl0}}\frac{\partial {\bar{u}}_{\theta l0}}{\partial \bar{z}}+\frac{1}{C_{l0}{\bar{u}}_{zl0}}\lambda_{zl}(R\omega -{\bar{u}}_{\theta l0})\right]+{\bar{u}}_{\theta l1}\cdot \left(-\frac{\lambda_{zl}}{C_{l0}}-i\frac{1}{R}\frac{{\bar{u}}_{\theta l0}}{{\bar{u}}_{zl0}}+i\frac{\Omega }{{\bar{u}}_{zl0}}\right)\\ \;-\;i\frac{1}{R\omega }\cdot \frac{{\bar{u}}_{zl0}^{2}}{R{\bar{u}}_{zl0}}\cdot {\bar{p}}_{l11}=\frac{1}{R\omega }\cdot \frac{R_{0}}{C_{l0}\varepsilon }\cdot \frac{\partial {\bar{u}}_{\theta l0}}{\partial \bar{z}},\\ \frac{\partial {\bar{p}}_{l1}}{\partial \bar{z}}+\frac{1}{\rho {\bar{u}}_{zl0}^{2}}R\omega \left[i\left(\Omega -\frac{{\bar{u}}_{\theta l0}}{R}\right)+\frac{2\rho \lambda_{zl}}{C_{l0}}{\bar{u}}_{zl0}\right]\cdot {\bar{u}}_{zl1}+i\frac{1}{\rho {\bar{u}}_{zl0}}{\bar{u}}_{\theta l1}\omega \\ \;=-\frac{R_{0}}{C_{l0}\varepsilon }\cdot \frac{\partial P_{l0}}{\partial \bar{z}}-i\frac{1}{\rho {\bar{u}}_{zl0}^{2}}\cdot \frac{R_{0}}{C_{l0}\varepsilon }\left(\Omega -\frac{{\bar{u}}_{\theta l0}}{R}\right), \end{array}\right.$$

where the non-dimensional first-order perturbation terms *ū_zl_*_1_, *ū_θl_*_1_, ${\bar{p}}_{{\;}_{l1}}$ are all functions of *z* and *t*, and λ*_zl_* is determined by Hirs' turbulent lubrication equations [42]. A series of numerical solutions for *ū_zl1_*, *ū_θl1_* and ${\bar{p}}_{{\;}_{l1}}$ can be acquired by solving equation (2.3) using the target method and the Newton–Raphson method combined with the three boundary conditions stated in the following equations:

2.4 $$\begin{array}{rl} & {\bar{p}}_{l1}{|}_{z=l}=0, \end{array}$$

2.5 $$\begin{array}{rl} & {\bar{u}}_{\theta l1}{|}_{z=0}=0 \end{array}$$

2.6 $$\begin{array}{rl} \;\mathrm{and}\; & {\bar{p}}_{l1}{|}_{z=0}=\frac{-R\omega (1+\xi_{z\mathrm{in}}){{\bar{u}}_{zl1}|}_{z=0}}{{\bar{u}}_{zl0}}. \end{array}$$

Thus, the first-order pressure distribution within the land portion along the axial direction can be obtained and expressed as

2.7 $${\bar{p}}_{l1}(z)=\left(\frac{r_{0}}{\varepsilon }\right)[\;f_{1c}(z)+if_{1s}(z)].$$

Similar to the analysis for the land portion, a series of non-dimensional first-order perturbation equations of the groove portion can be obtained as follows:

2.8 $$\left\{ \begin{array}{l} \frac{d{\bar{u}}_{zg1}}{dz}-i\frac{L_{g}}{R\cos\alpha }{\bar{u}}_{\theta g1}-i\frac{R_{0}}{T\varepsilon }\cdot \frac{L_{g}}{R\omega \cos\alpha }\left(\frac{\Omega }{T}-\frac{{\bar{u}}_{\theta l0}}{C_{l0}R}\right)=0,\\ \frac{d{\bar{u}}_{\theta g1}}{dz}\cdot \frac{\cos\alpha }{L_{g}}+\left\{\frac{1}{R\omega }\cdot \frac{1}{2T{\bar{u}}_{zg0}}[\lambda_{zg}-0.125\lambda_{f}{\bar{u}}_{\theta g0}]+i\left(\frac{\Omega }{{\bar{u}}_{\theta g0}R\omega }-\frac{1}{R^{2}\omega }\right)\right\}{\bar{u}}_{zg1}\\ -\left[\frac{{\bar{u}}_{zg0}}{2T}(\lambda_{zg}-0.25\lambda_{f})+i\left(\frac{1}{R}\frac{{\bar{u}}_{\theta g0}}{{\bar{u}}_{zg0}}-\frac{\Omega }{{\bar{u}}_{zg0}}\right)\right]{\bar{u}}_{\theta g1}-i\frac{1}{R\omega }\cdot \frac{{\bar{u}}_{zg0}^{2}}{R{\bar{u}}_{\theta g0}}{\bar{p}}_{g1}=0,\\ \frac{d{\bar{p}}_{g1}}{dz}+\frac{1}{\rho {\bar{u}}_{zg0}^{2}}{\bar{u}}_{zg1}R\omega \left[i\left(\Omega -\frac{{\bar{u}}_{\theta g0}}{R}\right)+\frac{\rho (\lambda_{zg}+0.25\lambda_{f})}{2T}{\bar{u}}_{zg0}\right]\\ \;+\frac{\omega }{\rho {\bar{u}}_{zg0}}{\bar{u}}_{g\theta 1}+\frac{R_{0}}{T\varepsilon }\cdot \frac{\partial P_{l0}}{\partial z}+i\frac{1}{\rho {\bar{u}}_{zg0}^{2}}\cdot \frac{R_{0}}{T\varepsilon }\left(\Omega -\frac{{\bar{u}}_{\theta g0}}{R}\right)=0, \end{array}\right.$$

where perturbation terms *ū_zg_*_1_, *ū_θg_*_1_, ${\bar{p}}_{g1}$ are all functions of *z* and *t*, and λ*_zg_* and λ*_f_* are determined by Hirs' turbulent lubrication theory [41]. A series of numerical solutions for *ū_zg_*_1_, *ū_θg_*_1_ and ${\bar{p}}_{g1}$ can be acquired by solving equation (2.8) combined with three boundary conditions stated as equations (2.9)–(2.11). Furthermore, the first-order pressure distribution within the groove portion along the axial direction can be obtained as equation (2.12),

2.9 $$\begin{array}{rl} & {\bar{p}}_{g1}{|}_{z=l}=0, \end{array}$$

2.10 $$\begin{array}{rl} & {\bar{u}}_{\theta g1}{|}_{z=0}=0, \end{array}$$

2.11 $$\begin{array}{rl} & {\bar{p}}_{g1}{|}_{z=0}=\frac{-R\omega (1+\xi_{z\mathrm{in}}){{\bar{u}}_{zg1}|}_{z=0}}{{\bar{u}}_{zg0}} \end{array}$$

2.12 $$\begin{array}{rl} \;\mathrm{and}\; & {\bar{p}}_{g1}(z)=\left(\frac{r_{0}}{\varepsilon }\right)[f_{2c}(z)+if_{2s}(z)]. \end{array}$$

Thus, the flow-induced force components within the land portions and the groove portions acting on the rotor due to shaft motion are represented as follows:

2.13 $$\begin{array}{rl} F_{x-\mathrm{sprial}}(t) & =F_{xl-\mathrm{spiral}}(t)+F_{xg-\mathrm{spiral}}(t)=-\varepsilon R\rho u_{zl0}^{2}\frac{L_{l}}{\cos\alpha }\sum_{n=1}^{Is}[(I_{s}\cdot L_{s}-1)\int_{\phi_{n}}^{\phi_{n+1/2}}\int_{0}^{1}f_{1c}(z)\cos\phi dzd\phi \\ & \;+\;I_{s}\cdot L_{s}\int_{\phi_{n+1/2}}^{\phi_{n+1}}\int_{0}^{1}f_{1c}(z)\cos\phi dzd\phi ]-\varepsilon R\frac{L_{g}}{\cos\alpha }\rho u_{{\;}_{zg0}}^{2}\sum_{n=1}^{Is}[(I_{s}\cdot L_{s}-1)\int_{\phi_{n}}^{\phi_{n+1/2}}\int_{0}^{1}f_{2c}(z)\cos\phi dzd\phi \\ & \;+I_{s}\cdot L_{s}\int_{\phi_{n+1/2}}^{\phi_{n+1}}\int_{0}^{1}f_{2c}(z)\cos\phi dzd\phi ] \end{array}$$

and

2.14 $$\begin{array}{rl} F_{y-\mathrm{spiral}}(t) & =F_{yl-\mathrm{spiral}}(t)+F_{yg-\mathrm{spiral}}(t)=-\varepsilon R\frac{L_{l}}{\cos\alpha }\rho u_{{\;}_{zl0}}^{2}\sum_{n=1}^{Is}[(I_{s}\cdot L_{s}-1)\int_{\phi_{n}}^{\phi_{n+1/2}}\int_{0}^{1}f_{1s}(z)\sin\phi dzd\phi \\ & \;+I_{s}\cdot L_{s}\int_{\phi_{n+1/2}}^{\phi_{n+1}}\int_{0}^{1}f_{1s}(z)\sin\phi dzd\phi ]-\varepsilon R\frac{L_{g}}{\cos\alpha }\rho u_{{\;}_{zg0}}^{2}\sum_{n=1}^{Is}[(I_{s}\cdot L_{s}-1)\int_{\phi_{n}}^{\phi_{n+1/2}}\int_{0}^{1}f_{2s}(z)\sin\phi dzd\phi \\ & \;+I_{s}\cdot L_{s}\int_{\phi_{n+1/2}}^{\phi_{n+1}}\int_{0}^{1}f_{2s}(z)\sin\phi dzd\phi ]. \end{array}$$

#### 2.3.2. Dynamic characteristics and forces of the plain part

The finite-length solution developed by Childs [2], which is widely used in industry, is applied for the dynamic analysis of the plain part in this paper. The flow-induced components within the middle plain part acting on the rotor due to shaft motion are defined by the following equations:

2.15 $$F_{x-\mathrm{plain}}(t)=\frac{-\varepsilon RL_{p}(P_{b1}-P_{b2})}{\lambda_{p}}\int_{0}^{1}\int_{0}^{2\pi }f_{3c}(z)\cos\phi dzd\phi$$

and

2.16 $$F_{y-\mathrm{plain}}(t)=\frac{-\varepsilon RL_{p}(P_{b1}-P_{b2})}{\lambda_{p}}\int_{0}^{1}\int_{0}^{2\pi }f_{3s}(z)\sin\phi dzd\phi .$$

#### 2.3.3. Dynamic characteristics and forces of the liquid annular seal with herringbone grooves

The dynamic forces and characteristics of the liquid seal with herringbone grooves are obtained by integrating those of the upstream spiral part, middle plain part and the downstream spiral part together. Hence, the dynamic forces within the seal can be stated as follows:

2.17 $$F_{x-\mathrm{herringbone}}(t)=F_{x-\mathrm{upspiral}}(t)+F_{x-\mathrm{plain}}(t)+F_{x-\mathrm{downspiral}}(t)$$

and

2.18 $$F_{y-\mathrm{herringbone}}(t)=F_{y-\mathrm{upspiral}}(t)+F_{y-\mathrm{plain}}(t)+F_{y-\mathrm{downspiral}}(t).$$

The definition of the flow-induced forces shown in equations (2.17) and (2.18) can be simplified by performing the integration at a time when the rotating displacement vector is pointing along the *X*-axis. At this time, *F_x_* and *F_y_* are both functions of the whirling velocity *Ω* for the reason that *f_c_* and *f_s_* are functions of *Ω*. Moreover, force components *F_x_* and *F_y_* can also be expressed as follows:

2.19 $$-F_{x-\mathrm{herringbone}}=\varepsilon [K_{h}+120\pi c_{h}\Omega -M_{h}{\left(120\pi \Omega \right)}^{2}]$$

and

2.20 $$-F_{y-\mathrm{herringbone}}=\varepsilon (k_{h}+120\pi C_{h}\Omega ).$$

Hence, the dynamic coefficients, including direct stiffness *K_h_*, cross-coupled stiffness *k_h_*, direct damping *C_h_*, cross-coupled damping *c_h_* and direct added mass *M_h_*, can be calculated by first evaluating equations (2.17) and (2.18) for the frequency set (*Ω*/*ω*: 0, 0.5, 1.0, 1.5, 2.0) combined with equations (2.19) and (2.20), and then performing a least-squares calculation. Furthermore, the dynamic forces and coefficients of the upstream grooved part depend on the inlet flow conditions of the seal and those of the next two parts depend on the circumferential velocity distribution of their upstream part.

### 2.4. Solution procedure

**Figure 3.**
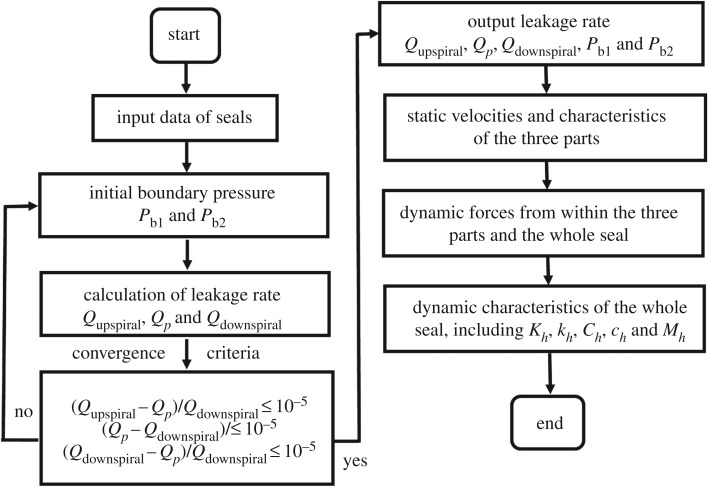
The entire calculation chart.

Figure 3 shows the entire calculation flow chart. As is shown, steady-state velocities and leakage rates of the two spiral-grooved parts and the middle plain part are determined first using the assumed initial boundary pressures. Then, an axial leakage equilibrium among the three parts is employed as the convergence criterion to determine the boundary pressures based on the zeroth-order perturbation results. The dynamic forces of the whole seal are obtained by integrating those of the three parts together. Furthermore, the rotordynamic characteristics of the seal including *K_h_*, *k_h_*, *C_h_*, *c_h_* and *M_h_* could be calculated by a least-squares method with five different frequency sets.

## 3. Results and discussion

### 3.1. Validation of the solution method

**Figure 4.**
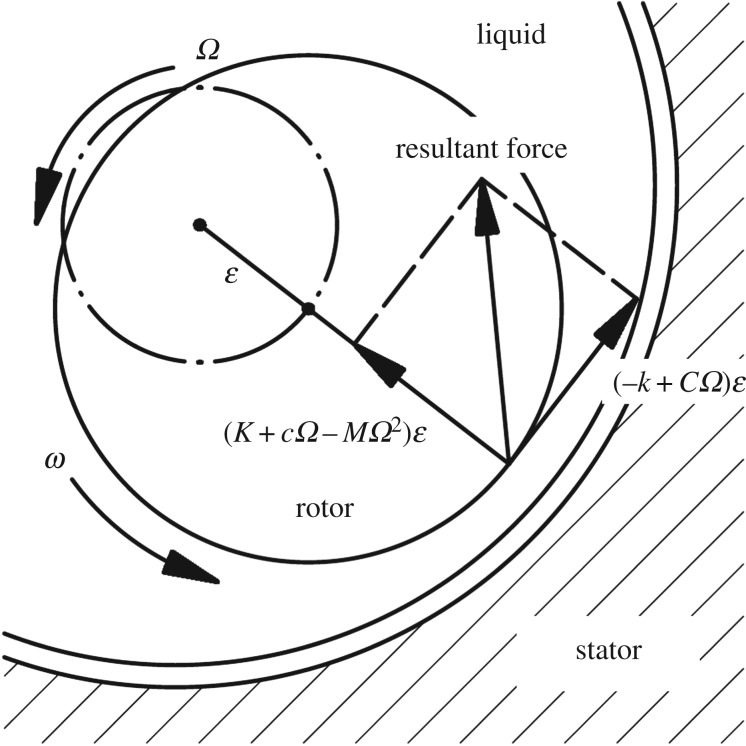
Whirling and rotating motions of a tested rotor and the forces on it.

Experiments for three sets of seals with herringbone grooves on the rotor were conducted on a specially designed test rig [25]. A forced whirling motion with a speed of *Ω* as shown in figure 4 was introduced in the experiments. The measured hydraulic forces *F_h_* are post-processed using an FFT filter to exclude the other forces such as the centrifugal force and gravity. Therefore, the validity of the presented approach is demonstrated by comparing the measured resultant forces and the predicted ones, which can be determined using equation (3.1). The three model seals have the same diameters, clearances, spiral angles, and land and groove depths and widths, but different lengths of each part (i.e. upstream spiral part, middle plain part and downstream spiral part), as listed in table 2. Table 3 illustrates the operating conditions and the identical geometric parameters of the seals,

3.1 $$F_{h}=\varepsilon \sqrt{{\left(K_{h}+c_{h}\Omega -M_{h}\Omega^{2}\right)}^{2}+{\left(-k_{h}+C_{h}\Omega \right)}^{2}}.$$

**Table 2.** Configurations of the model seals.

seal	length of the upstream spiral part (mm)	length of the middle plain part (mm)	length of the downstream spiral part (mm)	total length (mm)
1	4	4	4	12
2	8	4	8	20
5	12	4	12	28

**Table 3.** Operating conditions and geometric parameters of the tested model seals.

journal diameter (mm)	67	fluid density (kg m^−3^)	1000
radial clearance (mm)	0.5	dynamic viscosity (mPa s)	1.009
groove depth (mm)	0.5	whirling speed (r min^−1^)	672
groove width (mm)	1.5	pressure difference (kPa)	142
land width (mm)	1.5	rotating speed (r min^−1^)	360–2400
spiral angle (°)	3.97	number of threads	5

**Figure 5.**
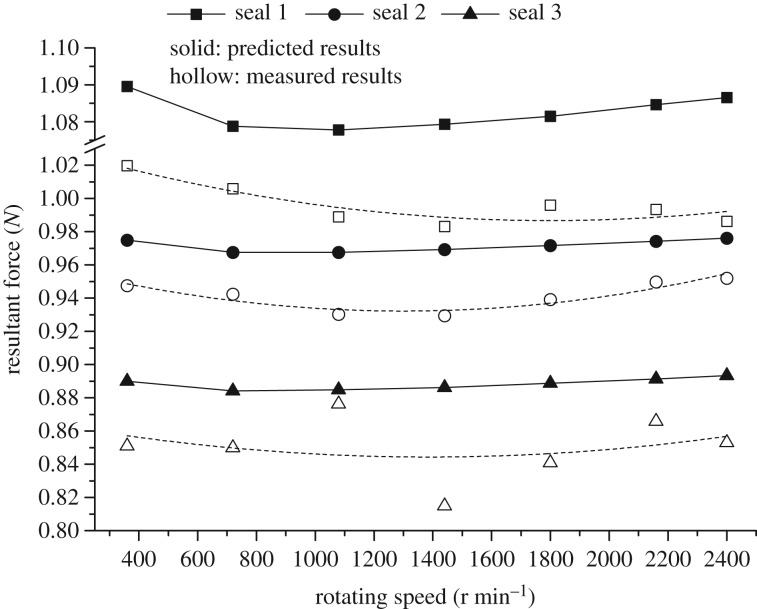
Comparisons between predicted and measured resultant forces.

Figure 5 compares the predicted and measured resultant forces of the three model seals under a series of rotational speeds. It illustrates that the changing trends of the predicted and experimental results are in accord. The prediction results have a margin of error of 11.15%. In general, the predicted results correlate well with the experimental evidence, which verifies the proposed calculation method and the analysis below based on it.

### 3.2. Effects of operating conditions on the leakage flow rate and dynamic characteristics

The effects of rotational speed and pressure difference on the leakage flow rates and dynamic characteristics of four sets of herringbone-grooved seals with different spiral angles were investigated using the proposed analysis method and compared with those of four spiral-grooved sets and one plain annular seal. The lengths of the nine calculation models were all 30 mm. As for the seals with herringbone grooves, the length of each part (i.e. upstream spiral part, middle plain part and downstream spiral part) was 10 mm. The other geometric parameters of the nine model seals used in this analysis, including journal diameter and dimensions of the grooves, are the same as those listed in table 3.

**Figure 6.**
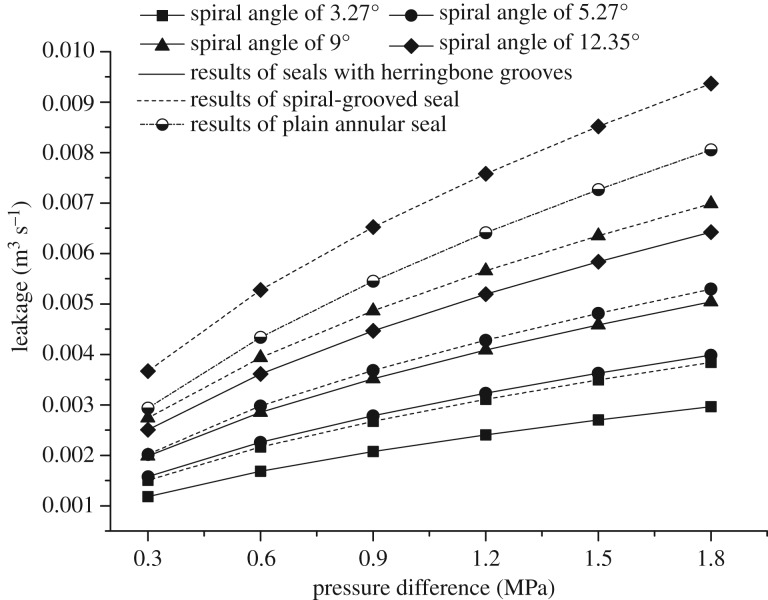
Leakage rate change with pressure difference.

Figure 6 shows the changes in the leakage flow rate with the pressure difference acting on the three kinds of seals at a rotational speed of 1000 r min^−1^. The leakage rates significantly increased with the pressure difference for all the seals. The leakage of most tested spiral-grooved and herringbone-grooved seals is much less than that of the plain seals under the same operating conditions. The herringbone-grooved seal model with the smallest spiral angle had the best sealing performance, while the spiral-grooved model with the largest angle had the worst. Compared with spiral grooves, herringbone grooves of the same spiral angle had a better sealing performance, especially those with small spiral angles.

Table 4 compares the leakage flow rates of the three types of seals at different running speeds ranging from 500 to 3500 r min^−1^ under a pressure of 1.0 MPa. It is observed that the leakage rate declines minimally with increasing speed almost for all the three seal types, and the leakage rate of the plain seal seems relatively more sensitive to the rotational speed than those of the other two kinds. Similar to the situation described in figure 6, leakage rates of both the herringbone-grooved and spiral-grooved seals show an upward trend with the increase in spiral angle. Additionally, it can be seen that the spiral angle has a greater impact on the leakage of the spiral-grooved seal than on that of the herringbone-grooved seal.

**Table 4.** Leakage rate of the three types of seals at different rotating speeds.

	leakage × 10^−3^ (m^3^ s^−1^)								
rotating speed (r min^−1^)	seals with herringbone grooves				spiral-grooved seals				plain seal
	3.27°	5.72°	9°	12.35°	3.27°	5.72°	9°	12.35°	
500	2.1922	2.9427	3.7190	4.7218	2.8267	3.8976	5.1427	6.8941	5.7878
1500	2.1909	2.9410	3.7169	4.7190	2.8265	3.8948	5.1416	6.8922	5.7841
2500	2.1893	2.9382	3.7104	4.7134	2.8260	3.8938	5.1396	6.8890	5.7768
3500	2.1851	2.9331	3.7032	4.7044	2.8254	3.8925	5.1370	6.8846	5.7660

According to the calculation results shown in figure 6 and table 4, the sealing performance of seals with herringbone grooves on the rotor is significantly better than that of spiral-grooved seals and plain seals, which will have a positive effect on the improvement of the single-stage head for centrifugal pumps. The leakage rate of seals with herringbone grooves depends more on the pressure difference but not so much on the rotational speed.

**Figure 7.**
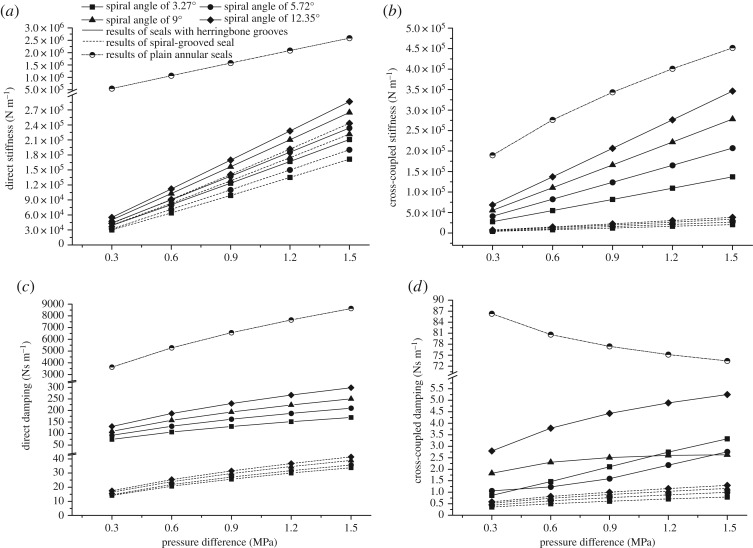
(*a*) Direct stiffness changes with pressure difference. (*b*) Cross-coupled stiffness changes with pressure difference. (*c*) Direct damping changes with pressure difference. (*d*) Cross-coupled damping changes with pressure difference.

Figure 7 demonstrates the effects of pressure difference on the rotordynamic coefficients of the seals described in §3.2. No comparison is made for the direct mass coefficient, because this term has little effect on the mass matrix of the whole shafting system. It is observed that the dynamic characteristics of seals with herringbone grooves depend more on the characteristics of the spiral-grooved parts due to the configurations. Nevertheless, this kind of seal combines the advantages of plain seals and spiral-grooved seals. The direct stiffness of herringbone-grooved seals is larger than that of spiral-grooved seals under the same operating conditions. A much smaller cross-coupled stiffness is achieved than with plain seals, which is good for the stability of the whole shafting system.

As for the dynamic characteristics of seals with herringbone grooves, it is indicated that direct damping and cross-coupled damping increase with the pressure difference acting on the seal. Similarly, direct stiffness and cross-coupled stiffness show a linear growth. Except for cross-coupled damping coefficients, the other coefficients of the seals with a smaller spiral angle are smaller than those with a bigger angle under the same operating conditions. Cross-coupled damping of the herringbone-grooved seal with a spiral angle of 9° shows a relatively smooth change compared with the other types and reaches a plateau at around 0.9 MPa.

### 3.3. Effects of seal configurations on the dynamic characteristics

**Figure 8.**
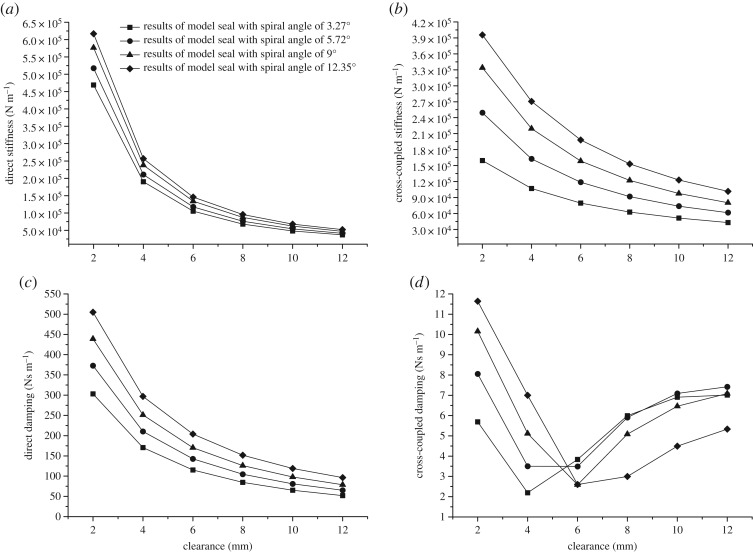
(*a*) Direct stiffness changes with clearance. (*b*) Cross-coupled stiffness changes with clearance. (*c*) Direct damping changes with clearance. (*d*) Cross-coupled damping changes with clearance.

Figure 8 presents the effects of the clearance value on rotordynamic coefficients. Direct stiffness, cross-coupled stiffness and direct damping decrease exponentially as the clearance increases, which is in accordance with the situation of plain annular seals. Cross-coupled damping plummets first and reaches the lowest point when the clearance is between 4 and 6 mm. Then, the cross-coupled damping coefficients increase gradually with the clearance value.

**Figure 9.**
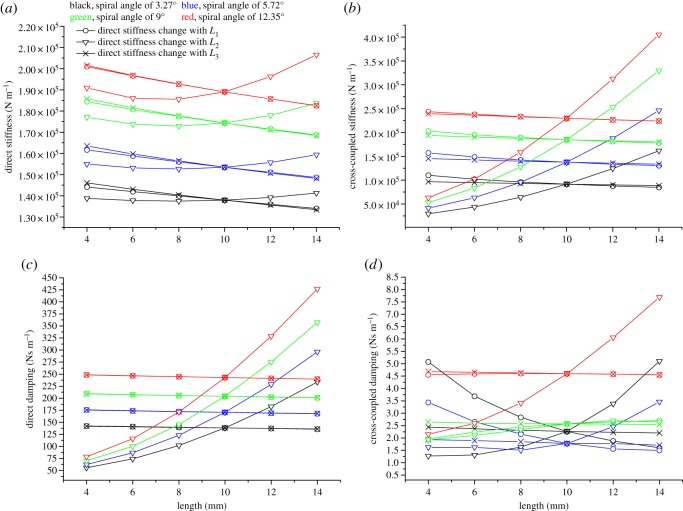
(*a*) Direct stiffness changes with the length of each part. (*b*) Cross-coupled stiffness changes with the length of each part. (*c*) Direct damping changes with the length of each part. (*d*) Cross-coupled damping changes with the length of each part.

Figure 9 illustrates the coefficient changes versus the lengths of the upstream spiral part, the middle plain part and the downstream spiral part, i.e. *L*_1_, *L*_2_, *L*_3_, respectively. A univariable analysis method is performed, which means that, when one of the three lengths is chosen as the variable, the other two stay equal to 10 mm. As is shown below, *L*_2_ is the key factor affecting rotordynamic coefficients. Direct stiffness decreases first with the increase of *L*_2_ and then increases, reaching a minimum at 8 mm. Cross-coupled stiffness and direct damping grow exponentially with *L*_2_. Cross-coupled damping of the seal with a spiral angle of 9° remains relatively stable with increasing *L*_2_, while those of the other kinds show an exponential growth, like cross-coupled stiffness and direct damping. Note that the changing rates for all the coefficients with *L*_2_ show an upward trend with an increase in the spiral angle. In most cases, the changes of direct stiffness, cross-coupled stiffness and direct damping with *L*_1_ are almost the same as those with *L*_3_. These coefficients decline linearly with the increase of *L*_1_ and *L*_3_. Meanwhile, the spiral angle has no effect on the declining rate of these coefficients, which is quite different from the situation of *L*_2_. It is shown in figure 6*d* that *L*_3_ has little effect on the cross-coupled damping coefficients. By contrast, cross-coupled damping changes with *L*_1_ are different for seals of different kinds. The damping coefficients of the seals with spiral angles of 3.27° and 5.72° decline substantially with *L*_1_, whereas those of the other two kinds change less.

## 4. Conclusion

A theoretical analysis method for calculating the dynamic characteristics of annular seals with herringbone grooves on the rotor is proposed and verified by comparisons with experimental results. With the proposed analysis method, the characteristics of this kind of seal under different operating conditions are predicted and compared with those of plain and spiral-grooved seals. The prediction results show that herringbone-grooved seals yield the lowest leakage flow among the three seal types. In addition, this kind of seal combines the advantage of plain seals (i.e. large direct stiffness) and the advantage of spiral-grooved seals (i.e. small cross-coupled stiffness) together, which will contribute to improving the rigidity and stability of the shafting system. Therefore, seals with herringbone grooves are attractive from the viewpoint of their superior static and dynamic characteristics.

Additionally, the influence of the seal geometric parameters, including the spiral angle, clearance value and lengths of the constituent parts, on the dynamic characteristics of herringbone-grooved seals is theoretically investigated. It is observed that most of the rotordynamic coefficients of this seal type with a bigger spiral angle are larger than those with smaller angles as a result of the increased leakage rate. As the lengths of the three parts, and especially the length of the middle plain part, significantly affect the dynamic characteristics, it is possible to optimize the constitutions and detailed parameters of herringbone-grooved seals for better sealing and rotordynamic performance.

## Data accessibility

Data are available from the Dryad Digital Repository (https://doi.org/10.5061/dryad.c2k7dj5) [43].
